# Supplementary material for: Identifying and prioritising climate change adaptation actions for greater one-horned rhinoceros (Rhinoceros unicornis) conservation in Nepal
Source: PeerJ. 2022 Jan 10;10:e12795. doi: 10.7717/peerj.12795 (PMC8757373; doi:10.7717/peerj.12795)
Supplement: Supplemental Information 1 [file peerj-10-12795-s001.docx]

**Coding for Key Informant Survey**

| ***Age group*** | |
| --- | --- |
| **1** | 18 to 30 years |
| **2** | 31 to 40 years |
| **3** | 40 to 50 years |
| **4** | >30 years |
| ***Gender*** | |
| **1** | Male |
| **2** | Female |
| ***Affiliation*** | |
| **1** | Government organisation |
| **2** | Non-government organisation |
| **3** | Community organisation |
| ***Experience*** | |
| **1** | <5 years |
| **2** | 5 to 15 years |
| **3** | >15 years |
| ***Perception on impacts of climate change*** | |
| **1** | Climate change impacts |
| **2** | Natural changes |
| **3** | Do not know |
| ***Perception on shift in habitat*** | |
| **1** | Habitat shift due to climate change |
| **2** | Habitat shift due to unknown reasons |
| **3** | Do not know |

**Coding for Stakeholder Consultation for Priority Ranking**

| ***Age group*** | |
| --- | --- |
| **1** | 18 to 30 years |
| **2** | 31 to 40 years |
| **3** | 40 to 50 years |
| **4** | >30 years |
| ***Gender*** | |
| **1** | Male |
| **2** | Female |
| ***Affiliation*** | |
| **1** | Government organisation |
| **2** | Non-government organisation |
| **3** | Community organisation |
| ***Experience*** | |
| **1** | <5 years |
| **2** | 5 to 15 years |
| **3** | >15 years |
| ***Adaptation actions*** | |
| **a** | Expand the existing protected areas |
| **b** | Establish new protected areas |
| **c** | Mange grasslands |
| **d** | Manage wetlands |
| **e** | Practice controlled burning |
| **f** | Control invasive species |
| **g** | Restore corridor and connectivity |
| **h** | Identify and protect climate refugia |
| **i** | Design and construct earthen mounds in floodplain grasslands |
| **j** | Conserve biodiversity at landscape level |
| **k** | Manage buffer zone |
| **l** | Prepare species conservation action plan |
| **m** | Integrate climate change impacts in species conservation action plan |
| **n** | Translocate species to other suitable habitats |
| **o** | Translocate species to future suitable habitat |
| **p** | Strengthen anti-poaching operation |
| **q** | Control water pollution |
| **r** | Mitigate human-wildlife conflicts |
| **s** | Conduct periodic census and Id-based monitoring |
| **t** | Initiate experimental research and monitoring of climate change effects |
| ***Priority score*** | |
| **0** | Not in priority |
| **9** | Highest priority |
